# Robotic Anatomical Liver Resection for Segment 7 Lesions Utilizing Saline-Linked Cautery (SLiC) Method

**DOI:** 10.7759/cureus.71537

**Published:** 2024-10-15

**Authors:** Takahisa Fujikawa, Yusuke Uemoto

**Affiliations:** 1 Surgery, Kokura Memorial Hospital, Kitakyushu, JPN

**Keywords:** intrahepatic glissonean approach, robotic anatomical liver resection, robotic right posterior sectionectomy, robotic segment 7 subsectionectomy, saline-linked cautery method

## Abstract

Introduction

Anatomical hepatectomy for segment 7 (S7) lesions is technically challenging due to their restricted accessibility and close proximity to the right hepatic vein, and the robotic approach for this challenging situation is currently not supported by conclusive data.

Methods

We present our novel technique of robotic anatomical hepatectomy for S7 lesions utilizing the saline-linked cautery (SLiC) method. Between 2022 and 2023, 10 robotic S7 subsectionectomy or right posterior sectionectomy were performed and included in the current study. The historical control group included patients undergoing laparoscopic anatomical hepatectomy for S7 lesions between 2017 and 2021 (n=9). Surgical outcomes were compared between the groups to assess the efficacy and safety of our technical robotic approach for S7 lesions.

Results

There were no conversions to open liver resection, no cases of grade B or C post-hepatectomy liver failure, and no mortality in the whole cohort. Although no difference was found between the laparoscopic and robotic groups in the difficulty score, operative time, and rate of red blood cell transfusion, the robotic group had a significantly lesser amount of surgical blood loss (28mL vs. 280mL, p=0.005). Concerning postoperative complications, one patient had liver subcapsular hematoma in the robotic group, although neither bile leakage nor intraperitoneal abscess occurred in the whole cohort.

Conclusions

Although robotic right posterior sectionectomy and S7 subsectionectomy of the liver are technically demanding procedures, the intrahepatic Glissonean approach using the SLiC method is safe and feasible. It might be performed without increasing the incidence of postoperative complications. Thus, the current approach can be considered as one of the preferred options for robotic anatomical hepatectomy for S7 lesions.

## Introduction

Anatomical liver resection (ALR) for segment 7 (S7) lesions is technically challenging due to their restricted accessibility and close proximity to the right hepatic vein (RHV) [1,2]. Laparoscopic liver resection (LLR), as well as an open hepatectomy, utilize several techniques for anatomical S7 subsectionectomy or right posterior sectionectomy using the intrahepatic Glissonean approach [1,3,4]. Nevertheless, the challenges posed by laparoscopic techniques, such as difficult exposure to the surgical field and limited range of motion of the device [5], make laparoscopic ALR for S7 lesions a complex procedure.

In the past few years, robotic liver resections (RLRs) have become widely accepted and expanded their range of uses. When comparing RLRs to traditional laparoscopic surgery, RLRs offer several benefits, including devices with seven-degree freedom, stable three-dimensional view, and filtration of tremors [6-8]. However, the process of liver parenchymal transection is particularly challenging during RLR due to the limited instruments that are necessary. In recent articles, the unique techniques of the saline-linked cautery (SLiC) method in RLR were described, in which either monopolar cautery scissors or bipolar cautery was used in conjunction with saline-dripping from the assistant’s side [9-11]. This technique enables both bleeding control and rapid transection of the liver tissue in RLR.

The current paper assessed the safety and feasibility of robotic ALR for S7 lesions via the intrahepatic Glissonean approach using the SLiC method.

## Materials and methods

Between September 2021 and December 2023, 82 RLRs were conducted at our institution, 10 of which were ALR for S7 lesions and were included in the current study (RLR-S7 group). Patients undergoing laparoscopic ALR for S7 lesions between January 2017 and July 2021 (n=9) were included in a historical control group (LLR-S7 group). Anatomical liver resection was defined as performing hepatectomy along the demarcation line after clamping the Glissonean pedicle. To assess the efficacy and safety of our technical robotic approach for S7 lesions, surgical outcomes were compared between the RLR- and LLR-S7 groups. The study protocol adhered to the principles outlined in the Declaration of Helsinki and received approval (#21021002) from the Kokura Memorial Hospital Clinical Research Ethics Committee.

Demographic information, surgical interventions, and postoperative results were obtained by systematically examining prospectively gathered databases and healthcare records. We evaluated surgical outcomes, as well as the practicability and safety of our procedure. We used the IWATE criteria [12], which ranks the difficulty level of RLR on a scale of 0-12. The Clavien-Dindo classification (CDC) [13] was used to categorize and evaluate postoperative complications; problems identified as CDC class II or higher were considered significant. Death occurring within 30 days following surgery was referred to as operative mortality.

Surgical techniques

All robotic procedures were performed using the da Vinci® Xi Surgical System, which is manufactured by Intuitive Surgical, Inc. (Sunnyvale, CA, USA). Preparation of the surgery and port placement were previously described [10,11]. Briefly, the patient was positioned in the left lateral decubitus position with the head slightly elevated by 8-10°. We usually employed five trocars for the procedure: robotic port #1 was positioned on the right upper lateral side of the abdomen, #3 was positioned above the umbilicus using Lap Protector™ with EZ Access™ (FF0707 or FF1010; Hakko Medical, Hongo, Tokyo, Japan), #2 and a 12-mm assistance port was placed between ports #1 and #3, and #4 was positioned on the left-sided epigastrium.

Intraabdominal pressure was maintained at 8 mmHg. Before parenchymal transection, the Pringle maneuver was prepared through the extraperitoneal tourniquet system in a standardized manner described in the previous study [14] and used on demand. We generally used monopolar curved scissors or Maryland bipolar forceps for dissection around the liver, whereas the SLiC method combined with saline dripping was exclusively performed during liver parenchymal transection [9-11]. For dissection around the liver, the Maryland bipolar forceps and fenestrated bipolar forceps were plugged into ForceTriad™ energy platform (Medtronic plc, Dublin, Ireland, Macro mode with an output of 70-80 W), and the integrated ERBE VIO dV (ERBE USA, Marietta, GA, Soft Coag mode with an effect 3 and power limit of 80 W), respectively. For the SLiC method using the monopolar curved scissors (SLiC-Scissors method), the scissors were plugged into the integrated ERBE VIO dV with Forced Coag mode (effect 1, power limit 40-50 W). In the case of the SLiC method, the bipolar forceps (Bipolar-SLiC method) were connected to the ForceTriad™ energy platform with a “standard mode” and an output of 40 W. 

**Figure 1:**
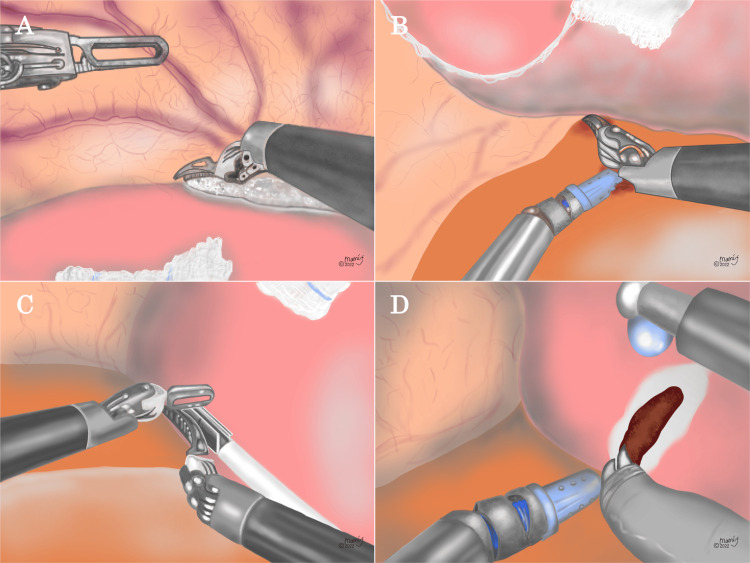
The process of full mobilization of the right liver in robotic anatomical liver resection for S7 lesions (A) The right coronary and triangular ligaments were dissected using the double bipolar method or monopolar curved scissors. (B) The liver was mobilized from the retroperitoneum. A dissection was made between the right adrenal gland and the liver. (C) After the right liver was fully mobilized, the Glissonean pedicle in S7 (G7), the venous branch in S7 (V7), and the hepatic tumor were identified by intraoperative ultrasound and marked. (D) The liver parenchymal transection was started just above the G7 using the saline-linked cautery (SLiC) method. Image Credits: Ms. Mamiko Fujikawa.

Figure 1 shows the process of full mobilization of the right liver in robotic ALR for S7 lesions. When the liver was mobilized, the Monopolar Curved Scissors® or Maryland Bipolar Forceps® was used. Following an extraperitoneal tourniquet device placement for the Pringle maneuver, the right coronary and triangular ligaments were dissected (Figure 1A; the round and falciform ligaments were generally preserved). Subsequently, the camera was moved from port #3 to #2 to secure the surgical field. Separation of the liver from the retroperitoneum was then performed to expose the inferior vena cava. Tip-up Forceps® in port #4 was used for liver retraction to stabilize the surgical field, and dissection was performed between the liver and retroperitoneum (Figure 1B). Before liver parenchymal transection, a hepatic tumor, Glissonean pedicle in the posterior portion (Gpost) and S7 (G7), the RHV, and a venous branch in S7 (V7) were observed using intraoperative ultrasound (Figure 1C). The liver parenchymal transection was started just above the G7 (Figure 1D).

**Figure 2:**
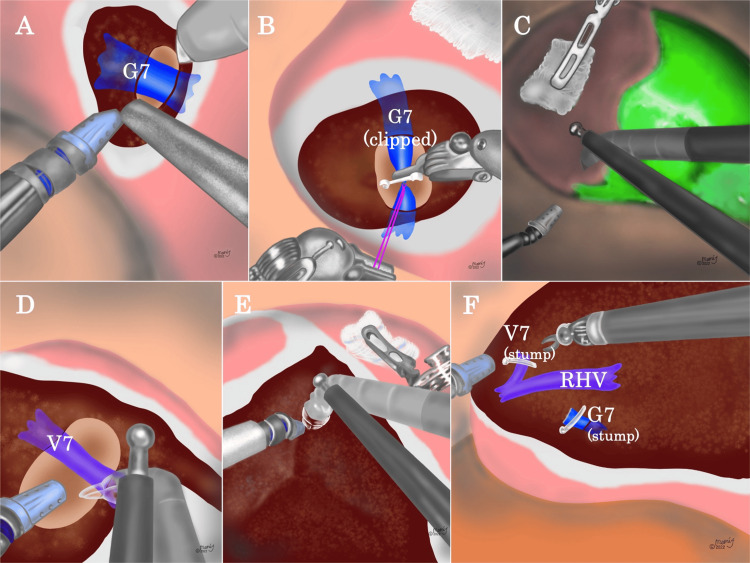
The process of liver parenchymal transection in robotic anatomical S7 subsectionectomy using the SLiC method (A) The root of the G7 was dissected using the SLiC method with bipolar cautery forceps (Bipolar-SLiC method) or the water-jet scalpel. (B) G7 was ligated and clipped. (C) The clear demarcation line for S7 was confirmed by intraoperative ICG negative staining. (D) V7 was exposed, clipped, and severed. (E) ICG imaging with the robotic FIREFLY system was used to correct the displacement of the cut plane as appropriate. (F) After the RHV was exposed from cranially to caudally, liver parenchymal transection was completed. SLiC, saline-linked cautery; G7, Glissonean pedicle in S7; ICG, indocyanine green; V7, the venous branch in S7; RHV: right hepatic vein. Image Credits: Ms. Mamiko Fujikawa

Figure 2 demonstrates the process of liver parenchymal transection in robotic anatomical S7 subsectionectomy using the SLiC method. During the liver parenchymal transection in RLR, either monopolar scissors (SLiC-Scissors) or bipolar cautery forceps (Bipolar-SLiC) were used [9, 11]. EndoWrist Suction Irrigator® in port #1 and Monopolar Curved Scissors® or Maryland Bipolar Forceps® in port #3 were used to transect the liver parenchyma. Using the SLiC method, hepatic parenchymal transection adjacent to the G7 was carefully performed (Figure 2A), and the root of the G7 was encircled, ligated, and clipped (Figure 2B). As a result, the S7 region became ischemic, and a definite demarcation border was detected, as confirmed by negative indocyanine green (ICG) staining (Figure 2C). Subsequently, V7 and RHV were exposed, and V7 was surrounded, clipped, and excised (Figure 2D). A negative ICG staining with the robotic Firefly system was used to appropriately correct the displacement of the excised plane (Figure 2E). Following RHV exposure from the cranial to the caudal portion, to reduce hemorrhage from the RHV, the liver parenchymal transection was completed (Figure 2F).

The procedures for robotic extended S7 subsectionectomy (S7 + dorsal side of S6 anatomical hepatectomy) and right posterior sectionectomy are outlined (Videos 1-2), respectively. Especially in the case of robotic right posterior sectionectomy, liver parenchymal resection around Gates V and VI, in accordance with Sugioka’s Gate Therapy [15], was performed in advance to avoid major vessel injury due to unintended movement of the robotics caused by loss of tactile sensation (Video 2). Additionally, this approach could minimize unnecessary dissection around the hepatic hilum and preserve the Glissonean branch of the paracaval portion in the caudate lobe (S1). Otherwise, liver mobilization and the method of liver parenchymal transection were mostly the same as those in robotic S7 subsectionectomy of the liver.

**Video 1:** The procedures for robotic extended S7 subsectionectomy (S7 + dorsal side of S6 anatomical hepatectomy) using saline-linked cautery (SLiC) method.

**Video 2:** The procedures for robotic right posterior sectionectomy utilizing saline-linked cautery (SLiC) method.

Statistics

Continuous variables were presented as median and range, whereas categorical variables were presented as absolute numbers and percentages. The Mann-Whitney U-test and Fisher’s exact probability test were used to compare continuous and categorical data. Two-sided P-values <0.05 were considered statistically significant. Statistical analyses were performed using EZR (Saitama Medical Center, Saitama, Japan), a GUI for R (version 2.13.0, R Foundation, Vienna, Austria) [16].

## Results

Patient and tumor characteristics of the participants are shown in Table 1. The RLR-S7 group included more patients with performance status 1 or 2 than the LLR-S7 group (90% vs. 44%, p=0.025), although there were no differences in the other background characteristics.

**Table 1:** Patient and tumor characteristics in the current cohort *Statistically significant LLR: laparoscopic liver resection; RLR: robotic liver resection; ASA: American Society of Anesthesiologists; PS: performance status; HCC: hepatocellular carcinoma; CRCLM: colorectal cancer liver metastasis; ICC: intrahepatic cholangiocarcinoma

Factors	LLR-S7 (n=9)	RLR-S7 (n=10)	p Value
Age, y, median (range)	77 (50-85)	70 (55-82)	0.287
Male sex, n (%)	6 (67%)	9 (90%)	0.303
ASA class 3 or higher, n (%)	3 (33%)	2 (20%)	0.550
PS 1 or 2, n (%)	4 (44%)	9 (90%)	0.025^*^
Liver cirrhosis, n (%)	2 (22%)	1 (10%)	0.582
Type of disease: HCC, n (%)	6 (67%)	5 (50%)	0.650
Type of disease: CRCLM, n (%)	2 (22%)	4 (40%)	0.629
Type of disease: ICC, n (%)	1 (11%)	1 (10%)	1.000
Size of the tumor, mm, median (range)	35 (22-75)	36 (22-60)	0.935

Table 2 shows the surgical factors and short-term outcomes of patients in the current cohort. There were no conversions to open liver resection, no cases of grade B or C post-hepatectomy liver failure, and no mortality in the whole cohort. Although no difference was found in the difficulty score, operative time, and rate of red blood cell transfusion, the RLR-S7 group had a significantly lesser amount of surgical blood loss than the LLR-S7 group (28 mL vs. 280 mL, p=0.005). Concerning postoperative complications, congestive heart failure occurred in one patient in the LLR-S7 group, and one patient had a liver subcapsular hematoma in the RLR-S7 group, although neither bile leakage nor intraperitoneal abscess occurred in the whole cohort.

**Table 2:** Surgical factors and outcomes in the current cohort *Statistically significant LLR: laparoscopic liver resection; RLR: robotic liver resection; RBC: red blood cell; PHLF: post-hepatectomy liver failure; LOS: length of postoperative stay; NA: not available

Factors	LLR-S7 (n=9)	RLR-S7 (n=10)	p Value
Procedure: S7 subsectionectomy, n (%)	1 (11%)	4 (40%)	0.303
Procedure: right posterior sectionectomy, n (%)	8 (89%)	6 (60%)	0.303
Difficulty Score, median (range)	11 (10-11)	11 (9-11)	0.215
Operative time, min, median (range)	505 (388-580)	492 (356-680)	0.806
Console time, min, median (range)	NA	418 (245-609)	NA
Blood loss, mL, median (range)	280 (30-1750)	28 (6-400)	0.005^*^
RBC transfusion, n (%)	1 (11%)	0 (0%)	0.474
Conversion to open surgery, n (%)	0 (0%)	0 (0%)	NA
PHLF (Grade B or C), n (%)	0 (0%)	0 (0%)	NA
Operative mortality, n (%)	0 (0%)	0 (0%)	NA
Postoperative complication, n (%)	1 (11%)	1 (10%)	1.000
LOS, d, median (range)	10 (8-25)	8 (7-13)	0.120

## Discussion

The current study described detailed technical aspects and short-term surgical outcomes of robotic ALR for S7 lesions using the SLiC method. This method allows quick and safe dissection around the major Glissonean pedicle and RHV during robotic ALR for S7 lesions. Most surgical outcomes after robotic ALR were comparable to those of laparoscopic ALR, and significantly shorter operative time was observed during robotic ALR than during laparoscopic ALR. Additionally, robotic ALR may be performed without increasing the incidence of postoperative complications, including bile leakage or bile duct stenosis in the right posterior section. Consequently, the current approach could deliver a safe and time-efficient approach for robotic right posterior sectionectomy or S7 subsectionectomy of the liver.

Various approaches, such as the intrahepatic Glissonean approach [1,3], the Glissonean approach from the hepatic hilu [17,18], or the caudate lobe−first approach, have been reported for laparoscopic surgery for anatomical right posterior segmentectomy and hepatic S7 subsegmentectomy [19,20]. However, except for the intrahepatic Glissonean approach, these approaches require unnecessary additional liver parenchymal resection around the hepatic hilum and/or caudate lobe, which can lead to unintended hemorrhage and/or biliary injury. The risk of major vessel injury due to loss of tactile sensation and concomitant unintentional movements can be disadvantages of robotic ALR, especially when using a robotic approach for ALR [8]. Given this rationale, we adopted a robotic intrahepatic Glissonean approach to minimize and avoid unnecessary dissection around the hepatic hilum. Additionally, utilizing the SLiC method during robotic ALR allows for more comfortable liver parenchymal transection around the major Glissons and hepatic veins using either monopolar scissors or bipolar cautery forceps [9,11]. Hence, we believe that the SLiC method employing the intrahepatic Glissonean approach is a highly recommended technique for performing robotic anatomical right posterior sectionectomy or S7 subsectionectomy of the liver.

The most serious technical issue in RLR is that the technique remains difficult to standardize due to a lack of instruments for liver parenchymal transection. A recent development in robotic liver surgery is called the SLiC method, which combines the benefits of laparoscopic and robotic surgical techniques, as detailed in a study [9,11]. This method is derived from the “Kyoto University-style liver parenchymal transection” technique used in open hepatectomy [21]. This involves applying low-temperature thermal coagulation below 100°C to the cut surface using a saline-linked bipolar electrocautery. By employing droplets of saline from the assistance side, the SLiC approach in RLR additionally makes use of superficial low-temperature thermal coagulation using monopolar cautery scissors (the SLiC-scissors) or bipolar cautery forceps (the Bipolar-SLiC) [9,11]. This procedure offers the advantage of keeping the tip of the scissors clean while allowing for fast, precise dissection and effective control of bleeding using saline. Using this strategy, rapid and secured dissection around the major Glissonean pedicle and RHV can be performed during robotic ALR for S7 lesions.

Regarding the SLiC method, we initially introduced the SLiC-Scissors method as a unique robotic technique for liver parenchymal transection using saline-linked monopolar scissors [9]. This approach achieves quick, thin-layered dissection and hemostasis while keeping the tip of the scissors clean with saline. In contrast, the Bipolar-SLiC method, which employs saline instillation in conjunction with clump-crushing, is challenging to handle with bleeding from the deep parenchymal fissure, resulting in a slower rate of liver parenchymal transection than that in the SLiC-Scissors method [11]. Nevertheless, the shape of the tip contributes to its advantageous nature, rendering it comparatively simple to operate and thus suitable for novices to handle. Furthermore, the bipolar cautery forceps have a more restricted spread of thermal coagulation than the monopolar cautery scissors, which reduces the possibility of thermal injury to deep tissue. As a result, during parenchymal dissection around the major Glissons, it can be employed safely during ALR.

Intercostal ports are beneficial for laparoscopic procedures targeting the posterosuperior region of the liver [2]. However, the EndoWrist® feature, which allows for manipulation of the instrument tips in seven different directions, can be used in robotic hepatectomy. The use of steady three-dimensional imaging and tremor filtration, along with the ability to scale motion, enables precise and comfortable dissections in robotic methods, even for complex anatomical S7 subsectionectomy or right posterior sectionectomy, without the need for intercostal ports.

This study has some limitations. Due to its retrospective nature, it is useful only in establishing the impact of treatments on outcomes. Furthermore, the sample size was inadequate, and a larger sample size would likely be advantageous in producing more reliable recommendations. However, we firmly believe our method significantly advances the eventual standardization of robotic ALR. It can potentially enhance safety and surgical outcomes and can be seen as a significant advancement towards the broad adoption of robotic ALR.

## Conclusions

Although robotic right posterior sectionectomy and S7 subsectionectomy of the liver are technically demanding procedures, the intrahepatic Glissonean approach using the SLiC method is safe and feasible. This technique facilitates the precise dissection and manipulation of the major hepatic vein and Glissonean pedicle located in the right posterior section. It might be performed without increasing the incidence of postoperative complications, including bile leakage or bile duct stenosis; thus, the current approach can be considered as one of the preferred options for robotic ALR for S7 lesions.
